# Humoral and Cellular Immune Responses to Vector, Mix-and-Match, or mRNA Vaccines against SARS-CoV-2 and the Relationship between the Two Immune Responses

**DOI:** 10.1128/spectrum.02495-21

**Published:** 2022-08-10

**Authors:** Minjeong Nam, Seung Gyu Yun, Sang-wook Kim, Chris Gunwoo Kim, Jae Hyun Cha, Cheonghwa Lee, Seunghyuk Kang, Seul Gi Park, Sun Bean Kim, Ki-Byung Lee, You-Seung Chung, Myung-Hyun Nam, Chang Kyu Lee, Yunjung Cho

**Affiliations:** a Department of Laboratory Medicine, Korea University College of Medicine, Korea University Anam Hospital, Seoul, South Korea; b Gyeryong City Health, Gyeryong-si, Chungcheongnam-do, South Korea; c Department of Laboratory Medicine, Korea University Anam Hospital, Seoul, South Korea; d Division of Infectious Diseases, Department of Internal Medicine, Korea University College of Medicine, Korea University Anam Hospital, Seoul, South Korea; Barcelona Centre for International Health Research (CRESIB, Hospital Clínic-Universitat de Barcelona)

**Keywords:** cellular immune responses, humoral immune responses, vector vaccine, mix-and-match vaccine, mRNA vaccine

## Abstract

We investigated how differences in age, sex, or vaccine type can affect humoral and cellular immune responses after vaccination with vector (ChAdOx1 nCoV-19), mix-and-match (first, ChAdOx1 nCoV-19, and second, BNT162b2), or mRNA (BNT162b2 or mRNA-1273) vaccines against severe acute respiratory syndrome coronavirus 2 (SARS-CoV-2). Venous blood was collected from 573 subjects (vector, 396; mix-and-match, 96; and mRNA, 81) before the first vaccination (*T*_0_), 7 to 8 weeks (vector) or 3 to 4 weeks (mRNA) after the first vaccination (*T*_1_), and 3 to 4 weeks after the second vaccination (*T*_2_). The humoral and cellular immune responses were evaluated using Elecsys anti-SARS-CoV-2 (Roche), Alinity SARS-CoV-2 IgG II Quant (Abbott), cPass SARS-CoV-2 neutralization antibody detection (GenScript), and QuantiFERON SARS-CoV-2 (Qiagen) kits. At *T*_1_, the levels of the receptor-binding domain antibodies (RBD Ab) and neutralizing antibodies (NAb) decreased with aging, but interferon gamma release (IGR) levels increased. The RBD Ab, NAb, and IGR levels were higher in females than in males at *T*_1_ and *T*_2_. The NAb levels were higher in the mix-and-match and mRNA vaccine groups than in the vector vaccine group at *T*_2_. The RBD Ab and IGR levels were higher in the mRNA vaccine group than in the vector or mix-and-match vaccine groups at *T*_2_. The optimal cutoffs for RBD Ab and NAb, which were used to determine the presence of T cell responses, were 5.7 binding antibody units per milliliter (BAU mL^−1^) and 12.0 IU mL^−1^, respectively. Age, sex, and vaccine type affected the humoral and cellular immune responses, and T cell responses could be estimated from RBD Ab and NAb levels.

**IMPORTANCE** There have been few studies that comprehensively evaluated factors affecting immune responses and the correlation between humoral and cellular immune responses after vector, mix-and-match, and mRNA vaccines against SARS-CoV-2. Therefore, we analyzed the effects of age, sex, and the different vaccine regimens on the immune responses to vaccination against SARS-CoV-2. The correlation between humoral and cellular immune responses and the cutoffs were derived for RBD antibodies and neutralizing antibodies to predict the presence of the cellular immune responses. In this comprehensive study, we demonstrated that there were differences in the immune responses induced after vaccination depending on the age and sex of an individual. Among the three vaccine regimens, the mix-and-match and mRNA vaccines induced the most robust immune responses. Finally, the proposed optimal cutoffs for RBD and neutralizing antibodies may be useful for predicting cellular immune responses when assays for cellular immune responses are not available.

## INTRODUCTION

Several vaccines have been developed rapidly to prevent and mitigate severe morbidity and mortality resulting from the coronavirus disease 2019 (COVID-19) pandemic, potentially enabling herd immunity in the future (1). The ChAdOx1 nCoV-19 (AstraZeneca, Cambridge, UK) vaccine is based on adenoviral vectors that encode the severe acute respiratory syndrome coronavirus 2 (SARS-CoV-2) spike (S) protein. The authorization recommends the administration of two doses at an interval of 4 to 12 weeks (https://www.who.int/publications/m/item/chadox1-s-recombinant-covid-19-vaccine). The BNT162b2 (Pfizer-BioNTech, Pfizer, Inc., NY, USA) vaccine and the mRNA-1273 (Moderna, Inc., Cambridge, MA, USA) vaccine contain mRNAs that encode the S protein of SARS-CoV-2 and are administered in two doses at intervals of 21 and 28 days, respectively (https://www.fda.gov/emergency-preparedness-and-response/counterterrorism-and-emerging-threats/coronavirus-disease-2019-covid-19). The South Korean government has approved the vaccination of South Koreans who were administered the first dose of ChAdOx1 nCoV-19 with BNT162b2 or mRNA-1273 as the second dose (mix-and-match vaccine) (https://ncv.kdca.go.kr/board.es?mid=a12101000000&bid=0031&act=view&list_no=558&tag=&nPage=1). In South Korea, 34.3% of individuals (17,788,268/51,812,669) were vaccinated with BNT162b2, 6.0% (3,019,692/51,812,669) with mRNA-1273, 20.9% (10,818,416/51,821,669) with ChAdOx1 nCoV-19, and 0.02% (9,053/51,812,669) with mix-and-match vaccine as of 16 October 2021 (https://ncv.kdca.go.kr/vaccineStatus.es?mid=a11710000000).

Humoral immune responses against SARS-CoV-2 are mediated by antibodies (Ab) against viral antigens, such as nucleocapsid (N) and S proteins, with a receptor-binding domain (RBD) (2). The presence of antibodies against N protein (N Ab) indicates previous infection. The presence of S Ab, RBD Ab, and/or neutralizing antibodies (NAb) indicates a prior infection with SARS-CoV-2 or a previous administration of COVID-19 vaccines (3). The activation of CD4^+^ T cells (helper T cells) and CD8^+^ T cells (cytotoxic T cells) represents the induction of cellular immune responses to previous infection or vaccinations (2). The vaccines have been designed to induce responses that skew T cell differentiation toward the interferon gamma (IFN-γ)-producing T helper 1 cell type (2).

Several previous studies have demonstrated that SARS-CoV-2 vaccines elicited robust humoral and cellular immune responses after vaccination (4–7). Other studies have investigated the effect of preexisting immunity, age, sex, vaccine type, genetic polymorphisms, underlying diseases, infection history, or a smoking habit on humoral immune responses (5, 8–18). The immune responses after vaccination with the most recently introduced mix-and-match vaccine have not been comprehensively analyzed. We aimed to demonstrate the effects of age, sex, and vaccine type (vector, mix-and-match, or mRNA vaccines) on humoral and cellular immune responses to SARS-CoV-2 vaccination.

Growing evidence suggests that the T cell responses against SARS-CoV-2 are essential and provide long-lasting immunity (19, 20). However, the commercial kits to monitor T cell responses for SARS-CoV-2 infection or vaccination are not widely available, and the humoral and cellular immune tests are not affordable in some medical insurance systems. Therefore, we have suggested cutoff levels of RBD Ab and NAb to determine the presence or absence of cellular immune responses from vaccinees’ data.

## RESULTS

### The effects of age, sex, and vaccine on the humoral and cellular immune responses to vaccination.

The median levels of RBD Ab and NAb after the first vaccination (*T*_1_; 7 to 8 weeks for vector or 3 to 4 weeks for mRNA vaccines) decreased significantly with age. Specifically, the median levels of RBD Ab and NAb were 73.3 binding antibody units per milliliter (BAU mL^−1^) and 201.6 IU mL^−1^, respectively, for subjects aged 20 to 29 years, 43.5 BAU mL^−1^ and 170.4 IU mL^−1^ for subjects aged 30 to 39 years, 42.2 BAU mL^−1^ and 207.5 IU mL^−1^ for subjects aged 40 to 49 years, 36.0 BAU mL^−1^ and 188.9 IU mL^−1^ for subjects aged 50 to 59 years, and 37.1 BAU mL^−1^ and 191.6 IU mL^−1^ for subjects aged ≥60 years (*P < *0.001) (Table 1). Conversely, the levels of interferon gamma release (IGR) at *T*_1_ showed age-dependent increases from 0.10 and 0.18 IU mL^−1^ to 0.40 and 0.60 IU mL^−1^ for CD4^+^ T cells and CD4^+^ T cells and CD8^+^ T cells, respectively (CD4^+^ T cells, *P < *0.001; CD4^+^ T cells and CD8^+^ T cells, *P = *0.001) (Table 1). However, there was no significant difference in the level of RBD Ab, NAb, or IGR 3 to 4 weeks after the second vaccination (*T*_2_).

**TABLE 1:** Humoral and cellular immune responses after vaccination based on age groups

Type of immune response (unit of measure), time point^*a*^	Median value (IQR) for indicated age group (yrs)^*b*^						*P* value
	20–29	30–39	40–49	50–59	≥60	Total	
RBD Ab (BAU mL^−1^)							
*T*_0_	—	—	—	—	—	—	
*T*_1_	73.3 (38.3–343.8)	43.5 (21.5–99.4)	42.2 (25.9–75.7)	36.0 (18.8–90.2)	37.1 (18.6–59.7)	45.5 (24.8–101.7)	<0.001^*c*^
*T*_2_	201.6 (90.3–1,154.1)	170.4 (98.3–455.3)	207.5 (86.1–589.9)	188.9 (79.4–712.2)	191.6 (71.9–607.3)	184.2 (88.6–609.2)	0.755
NAb (IU mL^−1^)							
*T*_0_	1.0 (0.0–4.0)	1.0 (0.0–4.0)	2.0 (0.0–6.0)	3.5 (0.0–8.5)	1.5 (0.0–7.0)	1.2 (0.0–6.2)	0.020
*T*_1_	76.5 (39.5–203.0)	48.0 (25.0–119.0)	36.0 (17.0–77.5)	33.0 (16.5–62.8)	34.0 (14.3–68.8)	45.7 (22.2–108.0)	<0.001^*d*^
*T*_2_	470 (179–1,468)	518 (149–1,338)	426 (121–1,859)	382 (120–1,507)	391 (91–1,512)	435 (141–1,539)	0.916
IGR (IU mL^−1^)							
CD4^+^ T cells							
*T*_0_	—	—	—	—	—	—	
*T*_1_	0.10 (0.04–0.31)	0.19 (0.07–0.46)	0.19 (0.06–0.51)	0.24 (0.06–0.74)	0.40 (0.14–0.95)	0.16 (0.05–0.51)	<0.001^*e*^
*T*_2_	0.18 (0.07–0.84)	0.37 (015–1.22)	0.53 (0.13–1.20)	0.42 (0.06–2.08)	0.40 (0.08–1.82)	0.29 (0.09–0.93)	0.283
CD4^+^ and CD8^+^ T cells							
*T*_0_	—	—	—	—	—	—	
*T*_1_	0.18 (0.07–0.53)	0.34 (0.11–0.85)	0.32 (0.13–0.93)	0.49 (0.12–1.43)	0.60 (0.13–1.38)	0.35 (0.08–1.22)	0.001^*f*^
*T*_2_	0.31 (0.12–1.53)	0.49 (0.22–1.54)	0.77 (0.20–2.19)	0.57 (0.07–2.94)	0.60 (0.08–1.83)	0.53 (0.12–1.84)	0.363

a RBD Ab, receptor-binding domain antibody; BAU mL^−1^, binding antibody units per milliliter; NAb, neutralizing antibody; IGR, interferon gamma release; CD, cluster of differentiation.

b IQR, interquartile range; —, the results are below the limit of detection. *n* = 573 participants.

c Subgroups with *P* < 0.05: 20 to 29 versus 30 to 39; 20 to 29 versus 40 to 49; 20 to 29 versus 50 to 59; 20 to 29 versus ≥60; 30 to 39 versus ≥60.

d Subgroups with *P* < 0.05: 20 to 29 vs.30 to 39; 20 to 29 versus 40 to 49; 20 to 29 versus 50 to 59; 20 to 29 versus ≥60; 30 to 39 versus 40 to 49; 30 to 39 versus 50 to 59; 30 to 39 versus ≥60.

e Subgroups with *P* < 0.05: 20 to 29 versus 40 to 49; 20 to 29 versus 50 to 59; 20 to 29 versus ≥60; 30 to 39 versus ≥60; 40 to 49 versus ≥60.

f Subgroups with *P* < 0.05: 20 to 29 versus 40 to 49; 20 to 29 versus 50 to 59; 20 to 29 versus ≥60.

The levels of RBD Ab, NAb, and IGR at *T*_1_ and *T*_2_ were significantly higher in females than in males (Table 2).

**TABLE 2:** Humoral and cellular immune responses after vaccination based on sex

Type of immune response (unit of measure), time point^*a*^	Median value (IQR) for indicated sex^*b*^		*P* value
	Male	Female	
RBD Ab (BAU mL^−1^)			
*T*_0_	—	—	
*T*_1_	57.4 (29.6–120.7)	36.8 (20.7–75.4)	<0.001
*T*_2_	209.5 (92.9–722.8)	146.1 (82.0–429.6)	0.009
NAb (IU mL^−1^)			
*T*_0_	1.0 (0.0–6.0)	1.0 (0.0–6.0)	0.417
*T*_1_	34.0 (16.0–76.0)	53.0 (27.0–126.0)	<0.001
*T*_2_	383 (122–1,492)	473 (160–1,573)	0.130
IGR (IU mL^−1^)			
CD4^+^ T cells			
*T*_0_	—	—	
*T*_1_	0.10 (0.03–0.30)	0.21 (0.07–0.65)	<0.001
*T*_2_	0.19 (0.06–1.11)	0.41 (0.10–1.45)	0.019
CD4^+^ and CD8^+^ T cells			
*T*_0_	—	—	
*T*_1_	0.17 (0.07–0.62)	0.38 (0.13–1.08)	<0.001
*T*_2_	0.37 (0.08–1.51)	0.62 (0.17–2.27)	0.023

a For abbreviations, see Table 1.

b —, result was below the limit of detection. *n* = 573 participants.

**FIG 1:**
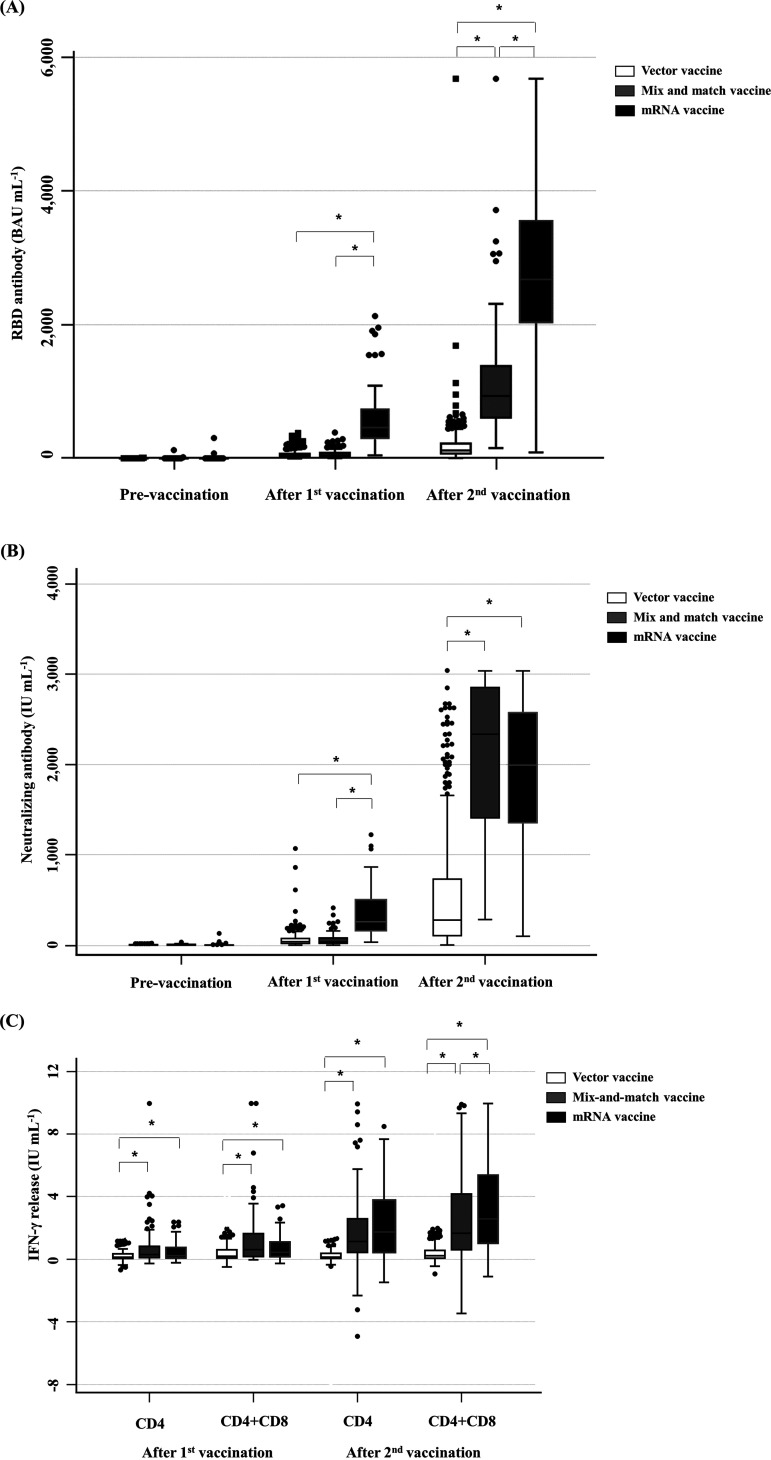
Humoral and cellular immune responses based on vaccine types (*n* = 573). RBD Ab (A), NAb (B), and IGR (C) levels were compared according to the vaccine types (vector, mix-and-match, or mRNA vaccines). RBD Ab, receptor-binding domain antibody; BAU mL^−1^, binding antibody units per milliliter; IFN-γ, interferon gamma; CD, cluster of differentiation. *, *P* < 0.05.

At *T*_1_, the median levels of RBD Ab and NAb were higher in the mRNA vaccine group than in the vector and mix-and-match vaccine groups; the RBD Ab levels were 39.9, 41.1, and 471.2 BAU mL^−1^ in vector, mix-and-match, and mRNA vaccine groups (*P < *0.001), respectively, and the NAb levels were 39.0, 37.0, and 281 IU mL^−1^ in vector, mix-and-match, and mRNA vaccine groups (*P* < 0.001), respectively. At *T*_2_, the median levels of RBD Ab were higher in the mRNA vaccine group than in the vector and mix-and-match vaccine groups (118.1, 933.7, and 2,649.7 BAU mL^−1^ in vector, mix-and-match, and mRNA vaccine groups, respectively; *P* < 0.001). The median levels of NAb at *T*_2_ were higher in the mix-and-match and mRNA vaccine groups than in the vector vaccine group (278, 2,338, and 1,995 IU mL^−1^ in vector, mix-and-match, and mRNA vaccine groups, respectively; *P < *0.001) (Fig. 1A and B). The levels of IGR at *T*_2_ in the mRNA vaccine group were higher than in the vector or mix-and-match vaccine groups, with levels of 0.13, 1.13, and 1.86 IU mL^−1^ for CD4^+^ T cells in vector, mix-and-match, and mRNA vaccine groups (*P* < 0.001), respectively, and 0.22, 1.66, and 2.56 IU mL^−1^ for CD4^+^ T cells and CD8^+^ T cells in vector, mix-and-match, and mRNA vaccine groups (*P* < 0.001), respectively (Fig. 1C).

Among the factors affecting the humoral and cellular immune responses, such as age, sex, and vaccine type, sex was an independent predictor of the humoral immune responses (odds ratio [OR] of 2.72; 95% confidence interval [CI], 1.04 to 7.13; *P = *0.04), and age was an independent predictor of the cellular immune responses (odds ratio of 9.95; 95% confidence interval, 2.72 to 36.37; *P = *0.001) (Table 3).

**TABLE 3:** Analysis of factors affecting humoral and cellular immune responses after vaccination

Immune response category, parameter	Value in indicated analysis^*a*^							
	Univariate				Multivariate			
	β	SE	*P* value	OR (95% CI)	β	SE	*P* value	OR (95% CI)
Humoral								
≥50 yrs old	0.80	0.47	0.09	2.21 (0.88–5.56)	0.88	0.48	0.07	2.42 (0.95–6.19)
Female	1.16	0.48	0.02	3.20 (1.24–8.27)	1.00	0.49	0.04	2.72 (1.04–7.13)
Type of vaccine								
Vector	−18.25	3,271	1.00	NA	−17.68	5,312	1.00	NA
Mix-and-match	−18.11	4,123	1.00	NA	0.85	6,648	1.00	NA
mRNA	18.02	5,371	1.00	NA	—	—	—	—
Cellular								
≥50 yrs old	2.16	0.58	< 0.001	8.68 (2.79–27.02)	2.30	0.66	0.001	9.95 (2.72–36.37)
Female	0.12	0.51	0.82	1.13 (0.41–3.08)	0.35	0.53	0.51	1.42 (0.5–4.02)
Type of vaccine								
Vector	−0.13	0.50	0.80	0.88 (0.33–2.33)	−0.01	1.17	1.00	1.00 (0.10–9.90)
Mix-and-match	−0.36	0.52	0.48	0.70 (0.25–1.91)	−0.34	1.26	0.79	0.71 (0.06–8.36)
mRNA	1.18	1.04	0.25	3.27 (0.43–25.08)	—	—	—	—

a β, beta coefficients; SE, standard error; OR, odds ratio; CI, confidence interval; NA, not available; —, in multivariate analyses, the mRNA vaccine was excluded due to high levels of variance inflation factor. *n* = 573 participants.

### A quantitative relationship between the humoral and cellular immune responses.

**FIG 2:**
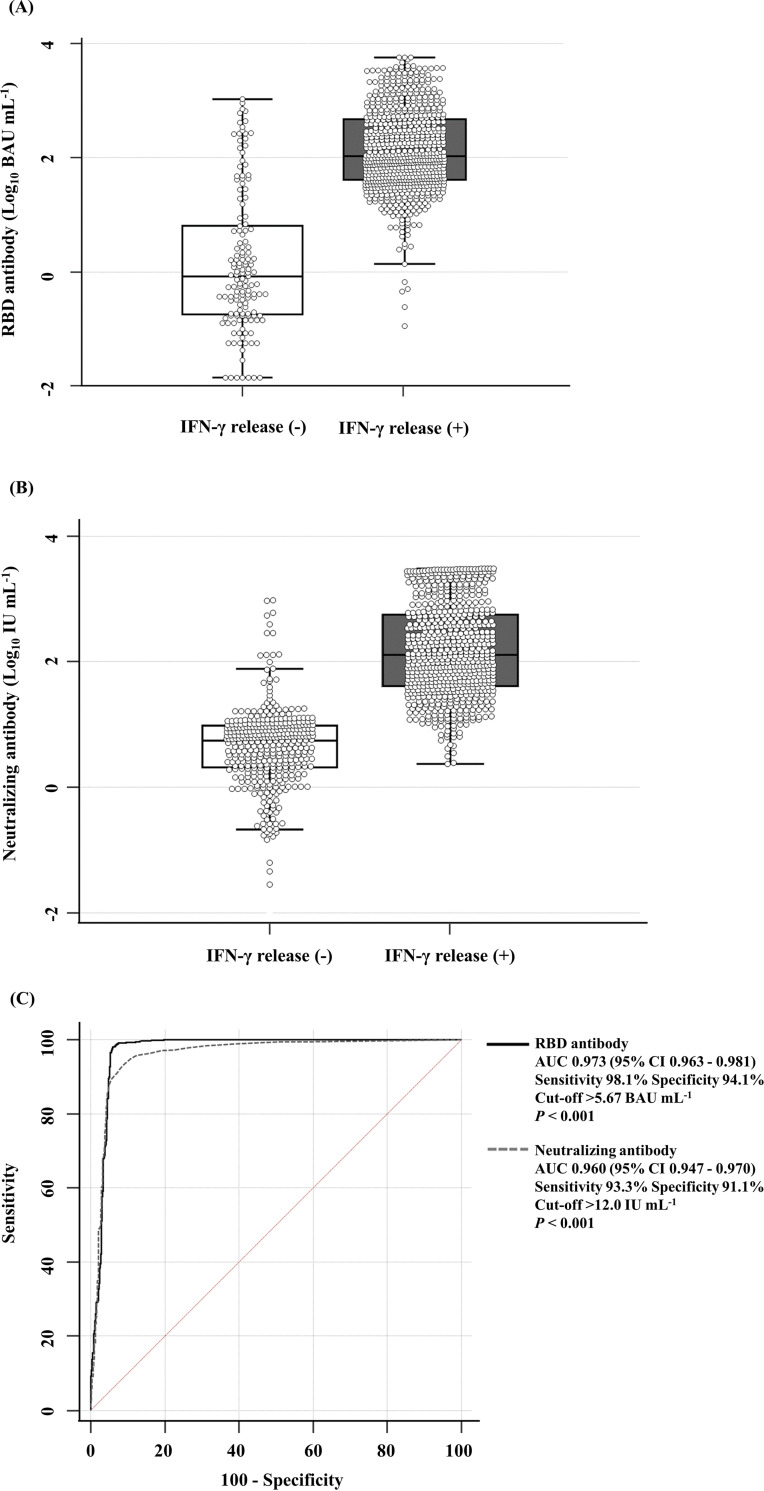
Comparison of RBD Ab and NAb levels based on results for IGR levels (*n* = 573). (A and B) The levels of RBD Ab (A) and NAb (B) are represented based on IGR-negative and -positive results for samples collected at *T*_0_, *T*_1_, and *T*_2_. In the box-and-whisker plot, the central box represents the values from 25th to 75th percentile, the middle line represents the median, and the vertical line extends from the minimum to the maximum value. (C) The optimized cutoff value to determine the presence of IGR was 5.7 BAU mL^−1^ for RBD Ab and 12.0 IU mL^−1^ for NAb. RBD Ab, receptor-binding domain antibody; BAU mL^−1^, binding antibody units per milliliter; IFN-γ, interferon gamma; AUC, area under the receiver operating characteristic (ROC) curve.

The levels of RBD Ab and IGR showed a moderate correlation with CD4^+^ T cells (ρ = 0.503, *P < *0.001) and a low correlation with CD4^+^ and CD8^+^ T cells (ρ = 0.498, *P < *0.001). NAb and IGR showed moderate correlation with CD4^+^ T cells (ρ = 0.597, *P < *0.001) and CD4^+^ and CD8^+^ T cells (ρ = 0.611, *P < *0.001) (Fig. S2 in the supplemental material). In the group with a negative IGR, the median levels of RBD Ab were −0.08 log BAU mL^−1^ (interquartile range [IQR], −0.74 to 0.81) (Fig. 2A) and the levels of NAb were 0.74 log IU mL^−1^ (IQR, 0.31 to 0.98) (Fig. 2B). In the group with a positive IGR, the median levels of RBD Ab were 2.03 log BAU mL^−1^ (IQR, 1.61 to 2.67) (Fig. 2A) and the levels of NAb were 2.10 log IU mL^−1^ (IQR, 1.61 to 2.74) (Fig. 2B). The optimized cutoff values for RBD Ab and NAb for the determination of IGR were >5.67 BAU mL^−1^ and >12.0 IU mL^−1^, respectively (Fig. 2C).

## DISCUSSION

This study investigated the humoral and cellular immune responses after the administration of vector, mix-and-match, or mRNA vaccines. We found differences in the humoral and cellular immune responses induced after vaccination by age, sex, and vaccine types. We also suggested optimized cutoff values for RBD Ab and NAb to determine the presence or absence of IGR.

Several factors contribute to the efficacy of immune responses to SARS-CoV-2 vaccines (8). In terms of age, many studies have demonstrated that increasing age leads to a decrease in the humoral immune responses to vaccination (9, 10). A previous study reported a decrease in the humoral immune responses with increasing age after the first vaccination with the ChAdOx1 nCoV-19 vaccine (RBD Ab with Abbott, 9,807, 5,496, and 4,156 arbitrary units [AU] mL^−1^, *P = *0.004, for subjects aged 18 to 55, 56 to 69, and ≥70 years, respectively). In contrast, there were no significant differences in the humoral immune responses after the second vaccination, regardless of age (*P = *0.68) (9). Another study showed that the humoral immune responses were lower in patients over 80 years of age than in those aged less than 60 years after the first or second vaccination with BNT162b2 (S Ab levels with Euroimmun after the first vaccination were 41.2 and 313 BAU mL^−1^, S Ab levels with Euroimmun after the second vaccination were 1,332.0 and 3,702.0 BAU mL^−1^, NAb values after the first vaccination were 1.2 and 16.1% inhibition, and NAb values after the second vaccination were 68.7 and 97.8% inhibition for subjects aged >80 and <60 years, respectively) (11). Our study results on the humoral immune responses were consistent with those of previous studies, and the age-dependent decline may be explained as immunosenescence.

The impact of immunosenescence on the cellular immune responses may vary depending on the age criteria. In a qualitative and quantitative evaluation, the cellular immune responses in the noninfectious vaccinated group were lower in the >60-years age group, whereas those in the infectious vaccinated group were higher in the >60-years age group (13). In another study, IGR was markedly decreased in the >80-years age group, and the <80-years age group did not show a statistically significant increase after the second vaccination compared to the increase after the first vaccination (14). However, our study showed an increase in cellular immune responses in the >50-years age group after the first vaccination only. Direct comparison with previous studies was not reasonable because of the differences in age category and study design. The cellular immune responses in the >50-years age group seemed to be affected by multiple factors, such as seasonal coronaviruses, underlying diseases, medication, smoking habits, previous exposure to SARS-CoV-2, and adverse reactions to vaccines. These factors could impact qualitative differences in CD4^+^ T cells by increasing or decreasing the age-dependent cellular immune responses rather than the participants experiencing a simple decline in T cell responses with aging (21).

Some studies have reported no differences in the humoral and cellular immune responses to vaccines based on the sex of an individual (5, 15). However, our study suggested that females had higher humoral and cellular immune responses than males. Sex can affect innate and adaptive immune responses, predisposition to autoimmunity, and vaccine efficacy (22, 23). This difference could be linked to the levels of hormones, such as estrogen and testosterone, which affect immune cell function (24).

The initial report from the United Kingdom Com-COV demonstrated that the reactogenicity of the mix-and-match vaccine group was higher than that of the vector vaccine group (ChAdOx1 nCoV-19) (25). An increasing number of studies have investigated the immunogenicity of the mix-and-match vaccine, and the mix-and-match vaccine induces robust humoral and cellular immune responses. The results have demonstrated increased humoral or cellular immune responses in the mix-and-match vaccine group or comparable immune responses between the mix-and-match and mRNA vaccine groups based on different assay platforms (5, 26–29). In this study, the RBD Ab levels in the mix-and-match vaccine group were lower than those in the mRNA vaccine group at *T*_2_. The reason for this is not clear at this time. However, one possibility is that, since the vector vaccines require a transcriptional step, the amount of S protein produced by the vector vaccine may be less than the amount induced by mRNA vaccine due to transcriptional errors. Alternatively, although we do not know exactly to what extent, the amino acid sequence of the S protein produced by the vector vaccine might be slightly different from that of the S protein induced by the mRNA vaccine due to the transcriptional step. From a B cell point of view, the difference could be large enough to create a different set of B cell receptor (BCR) repertoires between the vector vaccine and the mRNA vaccine. Therefore, two doses of the same mRNA vaccine may induce a stronger RBD antibody response than the mix-and-match vaccine, in which less S protein was produced at *T*_1_ or the same BCR repertoire was not stimulated at *T*_2_. Interestingly, there was no statistically significant difference in the NAb levels between the mix-and-match vaccine group and the mRNA vaccine group at *T*_2_. Since the NAb are the antibodies that neutralize the RBD-angiotensin-converting enzyme 2 (ACE2) interaction, the NAb also include antibodies other than those that can be detected with the RBD kit that detects antibodies that bind to the RBD. Therefore, we believe that the NAb level is a more desirable indicator of vaccine immunogenicity than the RBD Ab level. In addition, the mix-and-match vaccine may provide more protection against various future SARS-CoV-2 mutant infections than the other homogeneous vaccine regimens. However, additional studies are needed on breakthrough infections, hospitalizations, disease severity, or death rates in people vaccinated with different vaccine regimens before conclusions can be drawn. Although different conclusions can be drawn depending on the method used in the studies, our study showed robust immune responses in the mRNA and the mix-and-match vaccine groups.

CD4^+^ T cells help B cells produce antibodies. There were reports that the production of IgG RBD and interferon gamma (IFN-γ) was closely associated statistically (*r* = 0.6, *P* < 0.001) or that the IFN-γ release assay was useful for routine laboratories to evaluate cellular immunity against emerging variants of concern (VOCs) (30, 31). However, serological assays to monitor T cell responses against SARS-CoV-2 infection or vaccination are not widely available globally, including in South Korea. Therefore, we estimated the T cell immune responses from the RBD Ab and NAb levels of the vaccinees’ data and calculated the cutoff levels of RBD Ab (>5.7 BAU mL^−1^) and NAb (>12 IU mL^−1^) to determine the presence or absence of cellular immune responses. There were moderate correlations between RBD and IGR for CD4^+^ T cells (ρ = 0.503, *P* < 0.001) and between NAb and IGR (ρ = 0.597, *P* < 0.001) (Fig. S2).

Four of the excluded subjects deserve mention. One of the excluded subjects was confirmed positive for SARS-CoV-2 by reverse transcription-CR (RT-PCR) 11 weeks after the first vaccination; he had higher RBD Ab and NAb levels at *T*_2_ than those without infection histories. This was consistent with the findings of other studies (32, 33). Another excluded subject was not aware of being infected until the results from before the first vaccination (*T*_0_) were obtained, because of an absence of any symptoms; substantially high Ab levels at *T*_0_ were present for N Ab (cutoff index [COI] of 190), RBD Ab (1,060 BAU mL^−1^), and NAb (46 IU mL^−1^). The other two subjects did not have symptoms or signs of COVID-19, although the two were weakly positive for N Ab at *T*_0_, *T*_1_, and *T*_2_ (COI of 1.01 to 2.22) and positive for RBD Ab (153.2 and 8.3 BAU mL^−1^) and NAb (626 and 178 IU mL^−1^) at *T*_2_ only. We considered that the N Ab results were false positives due to contamination or cross-reactivity with seasonal coronaviruses (34).

This study has several limitations. First, the variables were not fully assessed because we did not review subjects’ clinical charts, perform RT-PCR at every visit, or ask about subjects’ smoking habits. However, all study subjects were asked about underlying diseases, medications, or previous exposure using a questionnaire before enrollment and at every sample draw. Second, the blood collection schedule for *T*_1_ differed depending on the vaccine regimen, creating difficulty in interpretation of *T*_1_ results. Third, the commercial RBD Ab kit is a semiquantitative assay and recognizes only the RBD, not the entire S protein contained in the vaccine. Therefore, the RBD Ab levels may not be quantitatively accurately measured or do not reflect the actual amounts of antibodies produced in those vaccinated.

Age, sex, and vaccine types seemed to affect immune responses in individuals. However, the mRNA vaccines and mix-and-match vaccines elicited the most robust immune responses and the vector vaccine elicited the lowest. Real-world conclusions on vaccine efficacy can be drawn from the breakthrough infection data. The RBD Ab test alone is not sufficient to evaluate the humoral vaccine immunogenicity exactly; the NAb test is additionally necessary. Our suggested optimal cutoffs for RBD Ab and NAb to predict the cellular immune responses could be useful in countries in which the IGR assay is not available.

## MATERIALS AND METHODS

### Study population.

This cross-sectional cohort study was conducted from March 2021 to September 2021 at the Korea University Anam Hospital (KUAH), Seoul, South Korea. A total of 612 subjects were enrolled, and we assessed the participants for current symptoms of COVID-19, a history of previous infection and contact with confirmed patients, adverse reactions after vaccination, and underlying diseases with a questionnaire before enrollment and at every blood draw. The subjects with prevaccination positive N Ab, RBD Ab, or NAb results were excluded. Reverse transcription-PCR (RT-PCR) was performed on subjects with clinically suspicious symptoms to confirm SARS-CoV-2 infection, and subjects with positive results were excluded. Five hundred seventy-three subjects were included in the study: 396 subjects were vaccinated with ChAdOx1 nCoV-19; 96 subjects with the mix-and-match vaccine; and 81 subjects with the BNT162b2 or mRNA-1273 vaccines. The characteristics of the study subjects are summarized in Table 4. The median ages of the individuals that received the vector, mix-and-match, or mRNA vaccine were 38 (interquartile range [IQR], 28 to 51), 51 (IQR, 45 to 58), and 27 (IQR, 26 to 30) years, respectively. Females were more prevalent in all groups (vector, 60.1%; mix-and-match, 62.2%; and mRNA, 80.2%). This study was approved by the Institutional Review Board of KUAH (K2021-0511-014), and informed consent was obtained from all subjects before sample collection. The Korea Disease Control and Prevention Agency recommends that the ChAdOx1 nCoV-19 vaccine be administered in two doses at an interval of between 8 and 12 weeks and that the BNT162b2 and mRNA-1273 vaccines be administered in two doses at an interval of between 3 and 4 weeks (https://www.korea.kr/news/pressReleaseView.do?newsId=156461314). Venous blood was collected from the subjects on three occasions: before the vaccination on the day of the first vaccination (*T*_0_), before the vaccination on the day of the second vaccination (*T*_1_), and 3 to 4 weeks after the second vaccination (*T*_2_). In the case of vector vaccine vaccinees, blood was collected 7 to 8 weeks after the first vaccination since the second vaccination was administered 7 to 8 weeks after the first vaccination. However, in the case of mRNA vaccine vaccinees, the second mRNA vaccines were administered 3 to 4 weeks after the first vaccination. Therefore, *T*_1_ sampling schedules differed depending on the vaccine regimen; blood was collected 7 to 8 weeks after the first vaccination for vector vaccinees and 3 to 4 weeks after the first vaccination for mRNA vaccinees. Three consecutive blood collections were obtained from 516 of the 573 participants; only partial blood sampling was possible from the rest (Fig. S1).

**TABLE 4:** Characteristics of the study population

Parameter, age group	Value [no. (%) or median (IQR)] for participants who received indicated type of vaccine^*a*^		
	Vector	Mix-and-match	mRNA
Total no.	396	96	81
Age (yrs)	37 (28–52)	51 (45–58)	27 (26–30)
Male	157 (39.6)	35 (36.5)	16 (19.8)
Female	239 (60.4)	61 (63.5)	65 (80.2)
20–29 yrs			
No.	124	2	57
Male	50 (40.3)	0 (0)	8 (14.0)
Female	74 (59.7)	2 (100)	49 (86.0)
30–39 yrs			
No.	88	13	19
Male	25 (28.4)	2 (15.4)	6 (31.6)
Female	63 (71.6)	11 (84.6)	13 (68.4)
40–49 yrs			
No.	77	28	5
Male	32 (41.6)	7 (25.0)	2 (40.0)
Female	45 (58.4)	21 (75.0)	3 (60.0)
50–59 yrs			
No.	64	35	0
Male	30 (46.9)	17 (48.6)	0 (0)
Female	34 (53.1)	18 (51.4)	0 (0)
≥60 yrs			
No.	43	18	0
Male	20 (46.5)	9 (50.0)	0 (0)
Female	23 (53.5)	9 (50.0)	0 (0)
Blood collection interval (days)			
1st^*b*^	57 (55–62)	42 (41–46)	25 (21–27)
2nd^*c*^	24 (20–29)	22 (21–24)	27 (25–28)

a IQR, interquartile range. *n* = 573 participants.

b Interval from 1st vaccination date to 1st postvaccination blood collection date (vector vaccine, 7 to 8 weeks; mRNA vaccine, 3 to 4 weeks).

c Interval from 2nd vaccination date to 2nd postvaccination blood collection date (all vaccines, 3 to 4 weeks).

### Blood collection.

Serum samples were used to assess the humoral responses and whole blood samples to assess the cellular responses. For assessment of the humoral responses, venous blood samples were collected in BD Vacutainer SST II advance tubes (stock keeping unit [SKU] 368640; Becton, Dickinson, Plymouth, UK) and immediately centrifuged at 2,300 × *g* for 10 min. Each serum sample was aliquoted into four tubes and stored at −80°C. For assessment of the cellular responses, 1-mL amounts of whole blood were directly collected into four QuantiFERON SARS-CoV-2 blood collection tubes. After 20 h of incubation at 37°C, the samples were centrifuged at 750 × *g* for 15 min and stored at 4°C until the test date.

### Assays for measuring the humoral immune responses to SARS-CoV-2.

The humoral immune responses were evaluated using Elecsys anti-SARS-CoV-2, Alinity SARS-CoV-2 IgG II Quant (Abbott Laboratories, Sligo, Ireland), and cPass SARS-CoV-2 neutralization antibody detection (GenScript, USA, Inc., NJ, USA) kits according to the manufacturer’s instructions.

The Elecsys anti-SARS-CoV-2 (N Ab) kit uses a recombinant antigen targeting the N protein and measures antibodies to N protein by using the double-antigen sandwich immunoassay. N Ab results were automatically calculated via a cutoff index (COI); a COI of <1.0 was considered nonreactive, and a COI of ≥1.0 was considered reactive. The manufacturer of the N Ab assay claims that the defined cutoff gives a sensitivity of 100% and a specificity of 99.8% for the detection of N Ab.

The Alinity SARS-CoV-2 IgG II Quant (RBD Ab) kit was designed to measure the IgG Ab against the RBD of the S1 subunit using the automated two-step sandwich Ab-binding immunoassay. The results for the RBD Ab are calculated automatically, with the antibody concentration represented using arbitrary units (AU mL^−1^). Binding antibody units per milliliter (BAU mL^−1^), which are traceable to WHO international standards for anti-SARS-CoV-2 immunoglobulin, were calculated using the following equation: BAU mL^−1^ = 0.142 × AU mL^−1^ (35).

The cPass SARS-CoV-2 neutralization detection kit is a competitive enzyme-linked immunosorbent assay that detects NAb against SARS-CoV-2. Samples premixed with horseradish peroxidase-labeled RBD were added to a 96-well plate precoated with the recombinant angiotensin-converting enzyme 2 (ACE2) receptors. After stopping the reaction with the substrate, the optical density (OD) was measured at 450 nm. Results were interpreted as the percentage of inhibition (% inhibition) using the following formula: % inhibition = (1 − OD value of sample/OD value of negative control) × 100. The percentage of inhibition results were then converted to IU mL^−1^ according to the formula proposed by the manufacturer to increase the comparability of the NAb results to those of other studies (36).

### Assays for measuring the cellular immune responses to SARS-CoV-2.

The cellular immune responses were evaluated with interferon gamma release (IGR) by QuantiFERON SARS-CoV-2 (research use only; Qiagen, Venlo, The Netherlands) using an enzyme-linked immunosorbent assay. One milliliter of venous blood was directly drawn into each of four QuantiFERON SARS-CoV-2 blood collection tubes (Qiagen): the nil tube, the Ag1 tube containing CD4^+^ T cell-specific epitopes from the S1 subunits, the Ag2 tube containing CD4^+^ T cell and CD8^+^ T cell-specific epitopes from the S1 and S2 subunits, and the mitogen tube. The nil and mitogen tubes were used to calculate the background signal and to act as negative and positive controls, respectively. IGR levels were calculated by subtracting the value of the nil tube from that of the Ag1 or Ag2 tube. IGR was considered positive if the IGR levels obtained after subtracting the prevaccination results from the first or second postvaccination results were greater than zero.

### Statistics.

The distribution normality and homogenous variation of all data were evaluated using the Kolmogorov-Smirnov test. Continuous variables with nonparametric distribution were represented as median values and IQRs, and categorical variables with nonparametric distribution were represented as numbers and percentages. The differences for age, sex, and vaccine types were analyzed using the Kruskal-Wallis test with a *post hoc* test. Univariate and multivariate logistic regression analyses were performed to identify predictors of humoral and cellular immune responses, such as age, sex, and vaccine types. The levels of RBD Ab and NAb were log transformed and compared with the IGR results. The cutoff values for RBD Ab and NAb IGR to determine the presence or absence of T cell responses were calculated using the Youden maximum index value with equal weightage to sensitivity and specificity. Since the sample size was not large, nonparametric tests were employed and correlations among the methods were analyzed by Spearman’s correlation coefficient (ρ). The two-sided 95% confidence interval (CI) was calculated. Statistical significance was set at a *P* value of <0.05. Statistical analyses were conducted using MedCalc version 20.014 (MedCalc Software Bvba, Ostend, Belgium) and SPSS version 25.0 (IBM Corp., Armonk, NY, USA).

### Data availability.

A data set of 577 subjects was deposited at https://dataverse.harvard.edu/ (https://doi.org/10.7910/DVN/YKC29Z).
