# Supplementary material for: Humoral and Cellular Immune Responses to Vector, Mix-and-Match, or mRNA Vaccines against SARS-CoV-2 and the Relationship between the Two Immune Responses
Source: Microbiol Spectr. 2022 Aug 10;10(4):e02495-21. doi: 10.1128/spectrum.02495-21 (PMC9431224; doi:10.1128/spectrum.02495-21)

Supplementary Figure 1. Flow chart of subject inclusion and exclusion criteria for the study.

Abbreviations: N, number; Ab, antibody.

Supplementary Figure 2. Correlation between RBD Ab and IGR and between nAb and IGR. Using Deming regression and Spearman correlation, (A) RBD Ab (BAU mL^{-1}) and IGR (IU mL^{-1}) were compared, and (B) nAb (IU mL^{-1}) and IGR (IU mL^{-1}) were compared. The black line represents the regression line.

Abbreviations: RBD Ab, receptor-binding domain antibody; IFN-γ, interferon-gamma; CD, cluster of differentiation

Supplementary figure 1

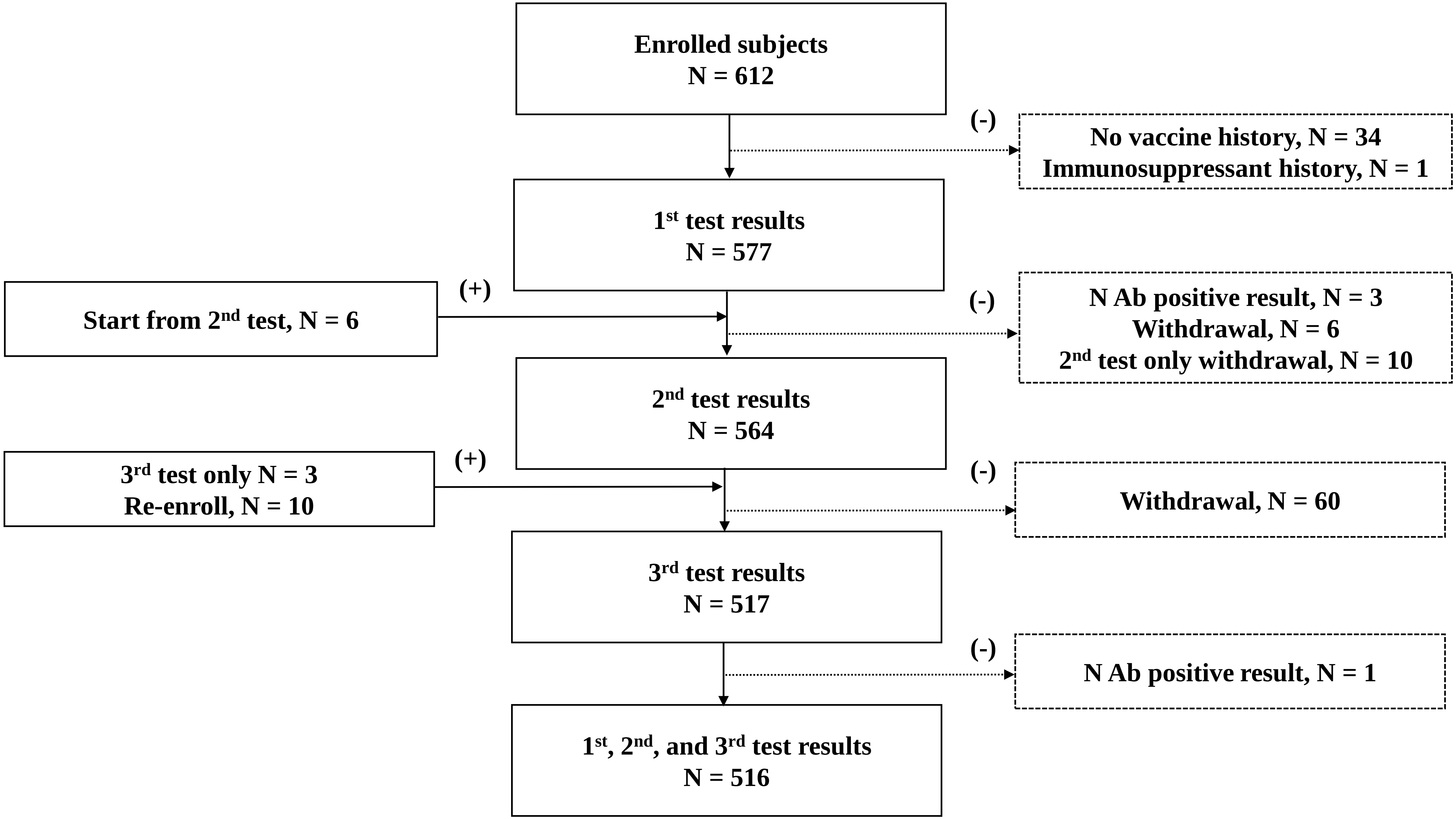

Supplementary figure 2-A

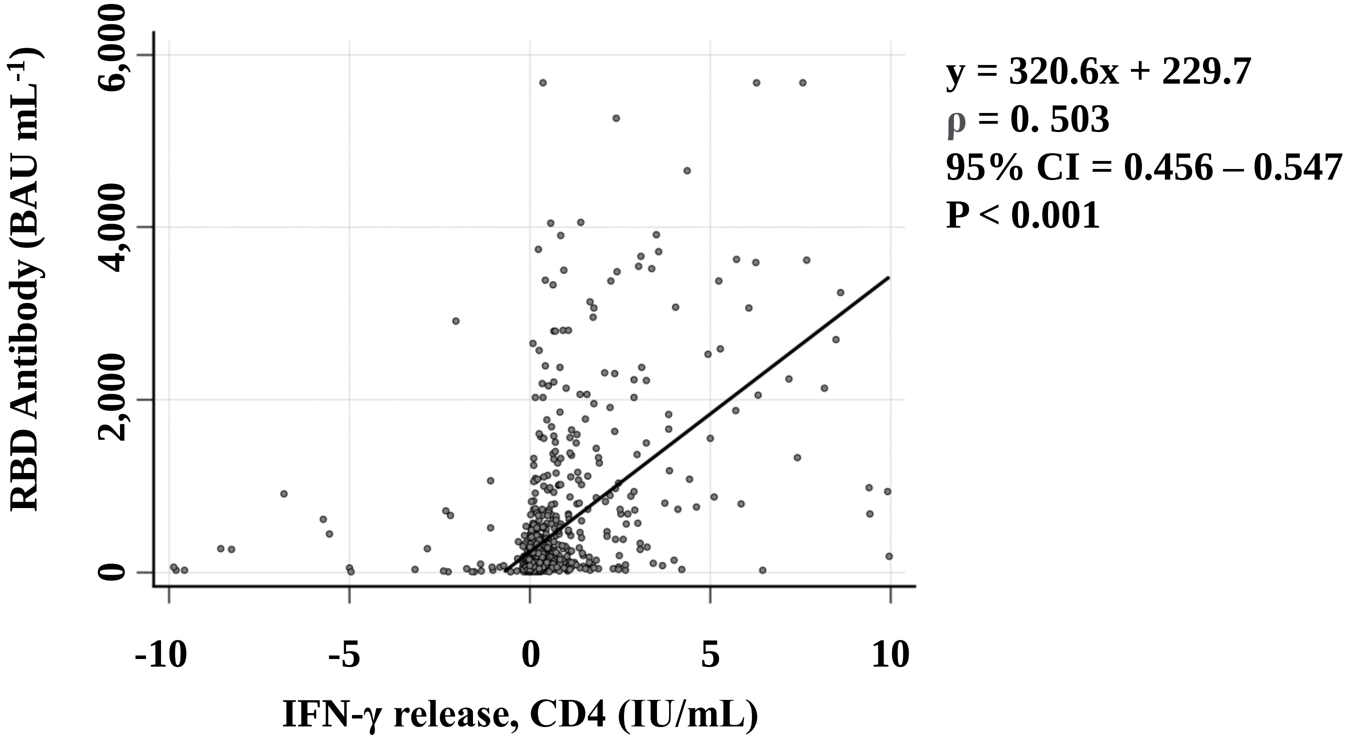

Supplementary figure 2-B

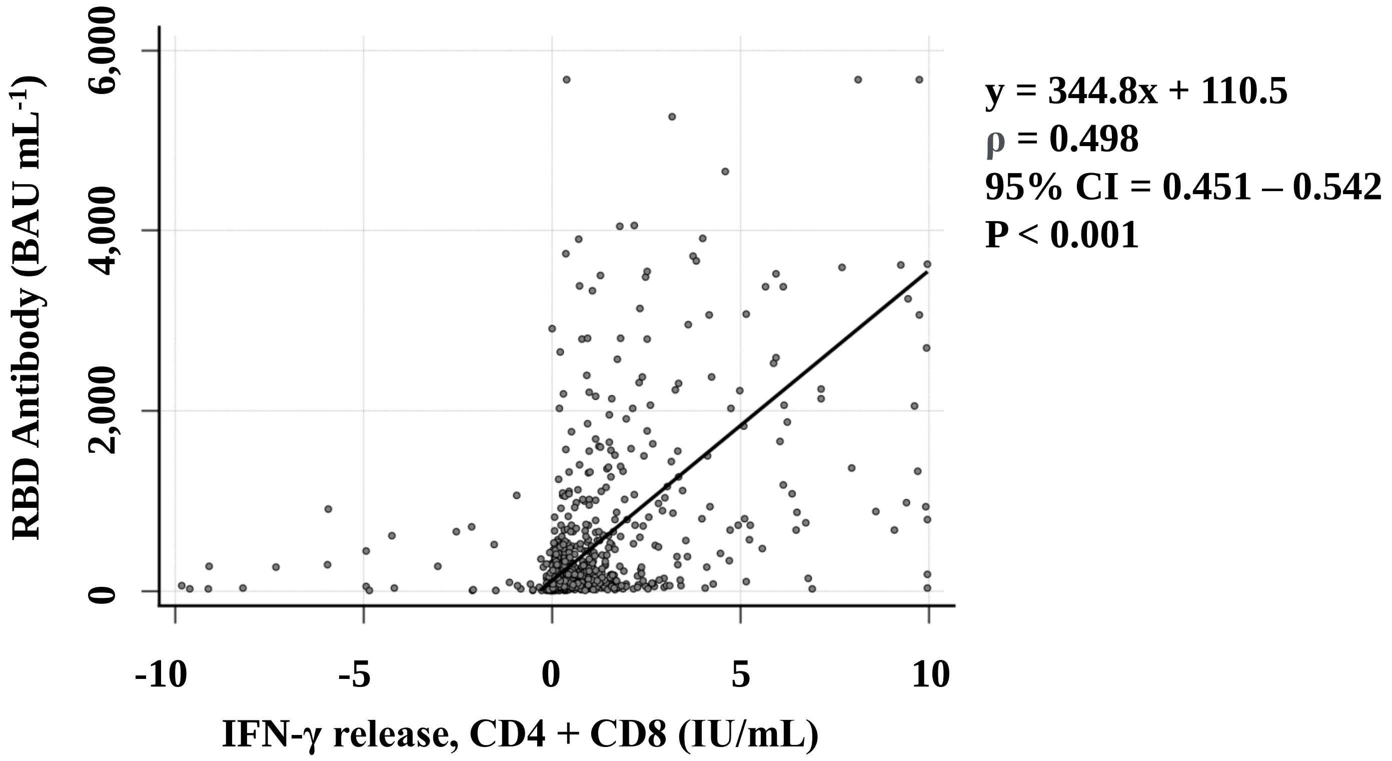

Supplementary figure 2-C

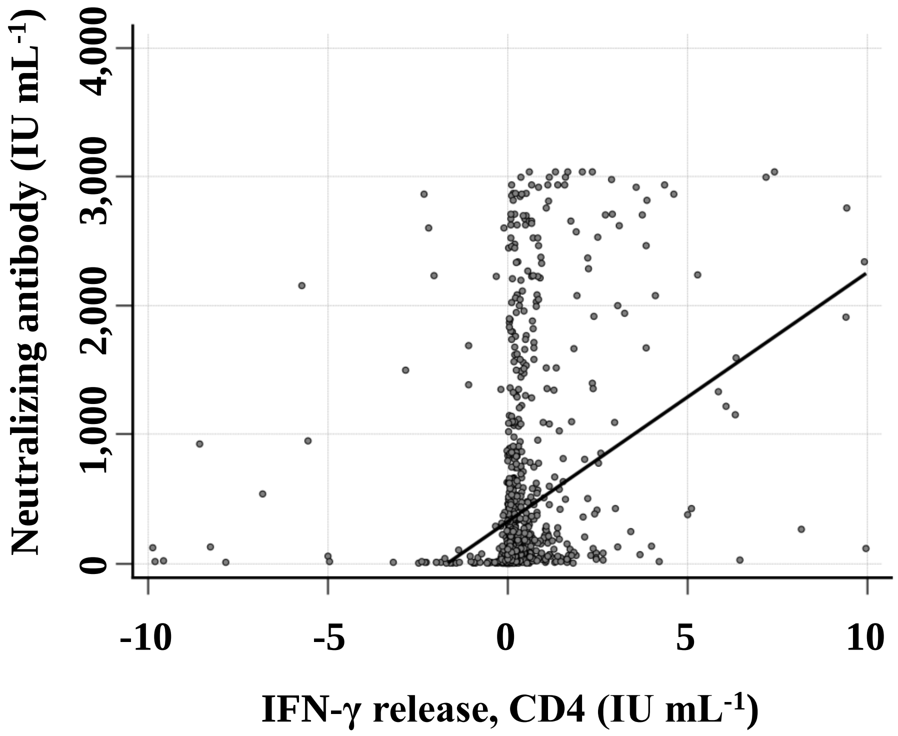

y = 193.5x + 323.2
ρ = 0.597
95% CI = 0.561 – 0.630
P < 0.001

Supplementary figure 2-D

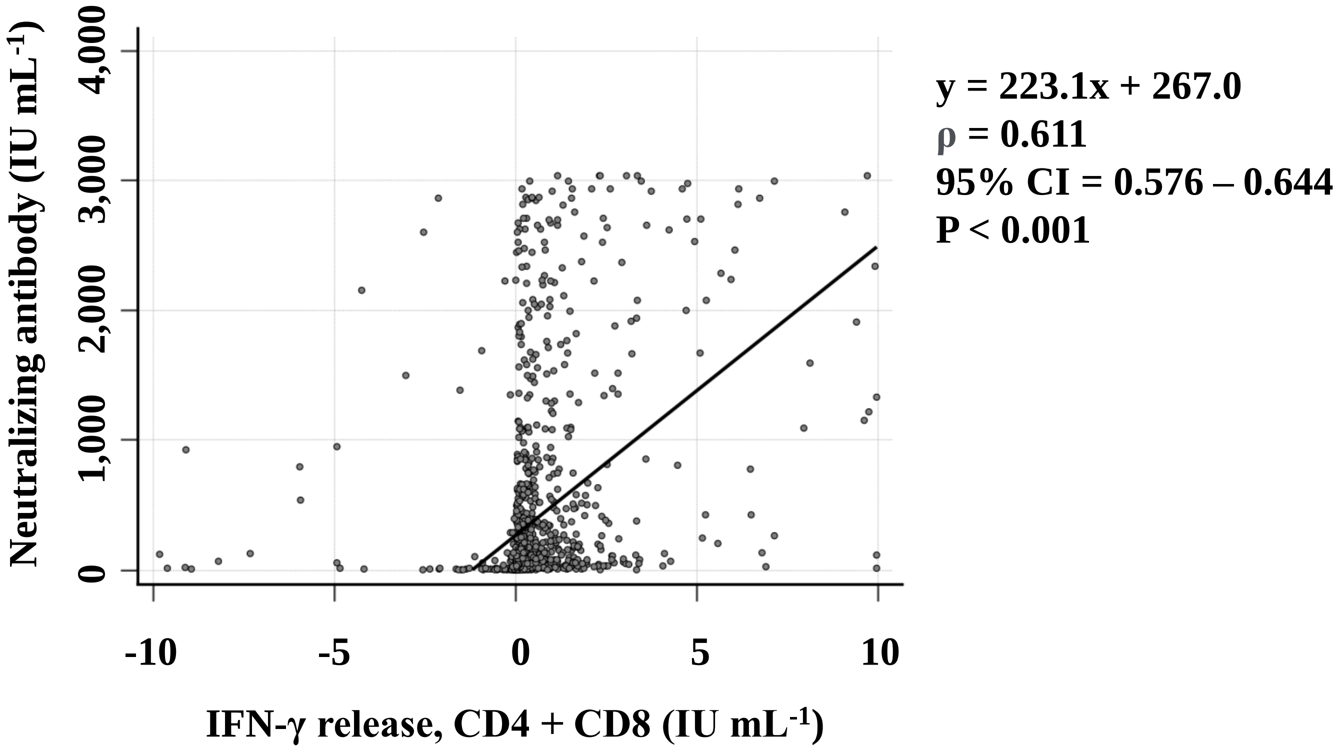

Supplement: Supplemental file 1 — Supplemental material. Download spectrum.02495-21-s0001.pdf, PDF file, 0.5 MB [file spectrum.02495-21-s0001.pdf]
